# Persistent expression of NLRP3 in spinal microglia promotes development of lumbar disc degeneration

**DOI:** 10.3389/fimmu.2022.1064303

**Published:** 2022-11-23

**Authors:** Peng Wang, Jing Zhang

**Affiliations:** Department of Orthopaedics, XinHua Hospital Affiliated to Shanghai Jiao Tong University School of Medicine, Shanghai, China

**Keywords:** lumbar disc degeneration (LDD), microglia, inflammation, PYD domains-containing protein 3 (NLRP3), neuropathic pain

## Abstract

**Introduction:**

Activated microglia play a critical role in the development of lumbar disc degeneration (LDD), which is a severe disease that causes neuropathic pain in affected people. Interleukin 1β (IL-1β) is a proinflammatory cytokine produced and secreted by activated microglia to induce the inflammation and the subsequent degradation of the disease discs. Recent findings suggest that activation of IL-1β in cells usually requires the involvement of NACHT, LRR and PYD domains-containing protein 3 (NLRP3)-induced formation of inflammasome. However, the importance of NLRP3 in spinal microglia in LDD is not known and thus addressed in the current study.

**Methods:**

NLRP3 expression was examined in the spinal discs. Correlation of NLRP3 levels in microglia with the pain score of the LDD patients or Thompson classification of the degeneration level of the patients was determined. The effects of persistent expression or depletion of NLRP3 on phagocytosis potential and production of proinflammatory cytokines in microglia were tested in vitro, while their effects on the severity of LDD and LDD-associated neuropathic pain were assessed in a mouse model for LDD.

**Results:**

NLRP3 was exclusively expressed in microglia in the spinal discs. NLRP3 levels in microglia strongly correlated with the pain score of the LDD patients, and modestly correlated with the Thompson classification of the degeneration level of the patients. Persistent NLRP3 expression in microglia increased both their phagocytosis potential and production of proinflammatory cytokines, while NLRP3-depleted microglia decreased both their phagocytosis potential and production of proinflammatory cytokines. In a mouse model for LDD, persistent NLRP3 activation in microglia significantly increased the severity of LDD and LDD-associated neuropathic pain, while specific depletion of NLRP3 in microglia significantly attenuated the severity of LDD and reduced the LDD-associated neuropathic pain.

**Conclusions:**

Persistent activation of NLRP3 in spinal microglia promotes development of LDD, while suppression of NLRP3 in microglia could be a promising strategy for LDD therapy.

## Introduction

Intervertebral disc degeneration (IVD) is the main cause of spinal degenerative diseases, and it causes a huge economic burden to the society due to the associated pain (1). The molecular mechanism of intervertebral disc degeneration has become the focus of research in recent years, as it is critical for understanding the whole process of IVD (2). During IVD, alterations in the extracellular matrix, inflammatory factors, and degrading enzymes in the intervertebral disc consisting of an outer layer of annulus fibrosus (AF), a middle layer of cartilage end plates and an inner layer of nucleus pulposus (NP) will trigger a cascade reaction, resulting in a pathological change to exhibit in morphology (3). Moreover, vascularization and innervation in the avascular discs occur, which leads to pain development (4). The methods for the treatment of intervertebral disc degeneration include ablation, surgical disc replacement and fusion. However, there is no effective treatment that can reverse the degeneration of the intervertebral disc nowadays (5).

Local neuroinflammation has been found to play a pivotal role in the initiation and progression of LDD (6). It is also believed that activated microglia and their production of pro-inflammatory cytokines are crucial for the neuroinflammation (7, 8). Previous studies have shown that conditioned media from degenerated discs activate microglia to a large extent to increase chemotaxis, migration, and release of pro-inflammatory mediators compared to normal discs (9, 10). However, the exact changes in microglia phenotype as well as any genes as key players during the process are not known.

Microglia are resident macrophages in the neural system. Like macrophages, microglia can either exhibit a pro-inflammatory phenotype, which is called “M1”, or an anti-inflammatory phenotype, which is called “M2”, is known as “polarization” (11). Interleukin 1β (IL-1β) is a cytokine that regulate both the progression and the severity of the inflammation. Moreover, IL-1β also regulate the phenotypic adaption and the function of antigen-presenting cells and immune cells such as neutrophils, macrophages and lymphocytes (12). Inflammasomes are the well-known activators for IL-1β (13). The most important inflammasome is PYD domains-containing protein 3 (NLRP3), which promotes the dimerization of caspase-1 to catalyze the pro-IL-1β into IL-1β to be active (14). However, a role of NLRP3 in microglia as well as its association with LDD has not been reported so far and thus addressed in the current study.

## Materials and methods

### Ethic approval

All the experiments including animal work and human studies have been approved by institutional Research Ethics Council from Shanghai Jiao Tong University School of Medicine. Previous written agreement was obtained from human participants who provided disc specimens.

### Animals

Several mouse strains were used to generate mice with microglia-specific depletion of NLRP3 or persistent activation of NLRP3 in the current study. A mouse with CreERT2 knock-in under the microglia-specific Tmem119 promoter (Tmem119p-CreERT2; #031820, Jax Mice, Bar Harbor, ME, USA) (15) was bred to a mouse with its NLRP3 exon 4 flanked by loxP sites (NLRP3 (fx/fx); #12935, Taconic Biosciences, Rensselaer, NY, USA) that allow constitutive knockout of NLRP3 in microglia after Cre-induced recombination (Tmem119p-CreERT2; NLRP3 (fx/fx)) induced by tamoxifen. A Tmem119p-CreERT2 mouse was also bred to a mouse with an insertion of a loxP-flanked neomycin resistance (neo) cassette in reverse orientation to the intron 2 of NLRP3 gene together with a missense mutation at exon 3, A350V, which corresponds to human amino acid 352 (NLRP3mut; #017969, Jax Mice) to generate a mouse with persistent activation of NLRP3 (due to the mutant NLRP3) in microglia (Tmem119p-CreERT2; NLRP3mut) after tamoxifen challenge. To induce occurrence of the Cre recombination, 1mg tamoxifen (Sigma-Aldrich, Shanghai, China) was daily and intraperitoneally injected to the mice for a consecutive 5 days. LDD was induced in 15-week-old mice by surgical removal of the spinal muscles, ligaments from supraspine and intraspine and posterolateral halves of the bilateral zygapophysial joints (16). Male and female mice were evenly distributed in all experimental groups. Group 1, NLRP3 (fx/fx) mice received sham operation (Sham); Group 2: NLRP3 (fx/fx) mice received LDD induction (LDD); Group 3: NLRP3mut mice received sham operation; Group 4: NLRP3mut mice received LDD induction; Group 5, Tmem119p-CreERT2; NLRP3 (fx/fx) mice received sham operation; Group 6: Tmem119p-CreERT2; NLRP3 (fx/fx) mice received LDD induction; Group 7: Tmem119p-CreERT2; NLRP3mut mice received sham operation; Group 8: Tmem119p-CreERT2; NLRP3mut mice received LDD induction. Mice were analyzed 8 weeks after LDD, or at age of 23-week-old. A von Frey filament test was performed as described before (16). Briefly, the mice were placed in a test box, from where Von Frey microfilaments were used to press the hind paw on the side of the mice to cause motionless withdrawal of its hind leg. Histological determination of LDD degrees was done using the method by Melgoza et al. (17).

### ELISA and immunostaining

Total protein was extracted from isolated cells or disc tissue, and then subjected to ELISA using specific kit for human NLRP3 (ab274401; Abcam, Cambridge, MA, USA) or mouse NLRP3 (ab279417; Abcam), IL-1β (ab197742; Abcam); tumor necrosis factor alpha (TNFα, ab208348; Abcam), interferon gamma (IFNɣ, ab282874; Abcam), arginase 1 (ARG1, ab269541; Abcam) and CD163 (ab272204; Abcam). Immunostaining for NLRP3 was done using a Rabbit specific HRP/DAB (ABC) Detection IHC Kit (ab64261; Abcam) with a rabbit anti-mouse NLRP3 antibody (ab272702; 1:75; Abcam).

### Flow cytometry

Spine discs from the mice were dissected out and dissociated into a single cell population with 45 minutes’ incubation with 0.25% Trypsin (Invitrogen, Carlsbad, CA, USA) at 37°C. The cells were then labeled with PE-cy5-conjugated CD68 and FTIC-conjugated Tmem119 antibodies (Becton-Dickinson Biosciences, Shanghai, China). Flow cytometry data were analyzed and were shown using FlowJo software (Flowjo LLC, Ashland, OR, USA).

### Analysis of phagocytosis

Phagocytosis was assessed based on 30 minutes’ zymosan intake by macrophages using a zymosan-based phagocytotic kit (ab211156, Abcam).

### Statistics and bioinformatics

For bioinformatics, single cell data from mouse spinal cords were obtained from an online public database, Panglaodb (www.panglaodb.se). Database numbers (SRS3059941: an adult sample, SRS3060017: a non-adult sample, SRS3059988: a non-adult sample, SRS3059989: a non-adult sample, SRS3059990: a non-adult sample, SRS3059991: a non-adult sample) from dataset (SRA667466) were used for this study. All data in this study were analyzed with GraphPad Prism 7 (GraphPad, Chicago, IL, USA) using one-way ANOVA with a Bonferroni correction for p value correction, after which a Tukey method was applied for comparison between two subgroups, since the sample number in each subgroup was equal. The individual values together with the mean and the standard deviation (SD) were shown in the figures. A p<0.05 was regarded as significance. P>0.05 was noted as non-significant (ns).

## Results

### Disc NLRP3 levels correlate with the pain and disc degeneration level in LDD patients

**Figure 1:**
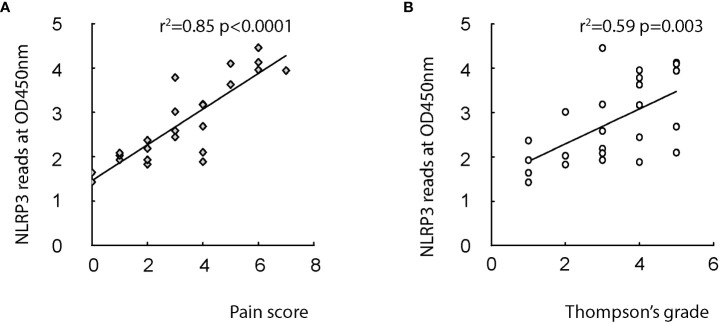
Disc NLRP3 levels correlate with the pain and disc degeneration level in LDD patients. Disc specimens from 24 participants who had different levels of Thompson classification of the degeneration and scores for pain were analyzed. **(A, B)** The NLRP3 protein levels in disc tissue were quantified by ELISA in all 24 specimens. Reads of NLRP3 OD values at 450nm were presented. The correlation between NLRP3 protein levels and pain score (**A**, r^2^ = 0.85, p<0.0001) or Thompson classification of the degeneration level (**B**, p=0.003) was assessed.

Microglia have been shown to play a critical role in the development of LDD. While the molecular regulation of microglia in the LDD setting is largely unknown. To address this question, we analyzed disc specimens from 24 participants who had different levels of Thompson classification of the degeneration and scores for pain (**Table 1**). We analyzed several candidate factors that are associated with regulation of microglia phenotype. Among these factors, we found that NLRP3, a regulator for conversion of pro-IL-1β into IL-1β, was very strongly correlated with patients’ pain score (ɣ^2^ = 0.85; p<0.0001; **Figure 1A**) and was modestly correlated with patients’ Thompson classification of the degeneration level (ɣ^2^ = 0.59; p=0.003; **Figure 1B**). These data suggest that NLRP3 may play a role in the LDD-associated pain.

**Table 1:** Demographic information of Intervertebral disc donors.

Sample	Sex	Age (years)	Thompson’s grade	Pain Score	Disc NLRP3 level readout at OD450
1	F	41	I	0	1.43
2	F	39	I	0	1.64
3	M	38	I	1	1.93
4	M	42	I	2	2.37
5	F	51	II	1	2.03
6	M	52	II	2	1.83
7	M	48	II	3	3.01
8	M	64	III	3	2.59
9	M	42	III	4	3.19
10	M	49	III	2	1.93
11	F	52	III	2	2.19
12	F	55	III	1	2.09
13	M	48	III	6	4.46
14	F	35	IV	3	3.79
15	F	56	IV	4	1.89
16	M	46	IV	6	3.96
17	F	34	IV	3	2.44
18	M	58	IV	5	3.63
19	F	47	IV	4	3.17
20	F	39	V	4	2.69
21	M	42	V	7	3.94
22	M	67	V	6	4.13
23	M	59	V	5	4.10
24	M	63	V	4	2.10

### NLRP3 is exclusively expressed in disc microglia

**Figure 2:**
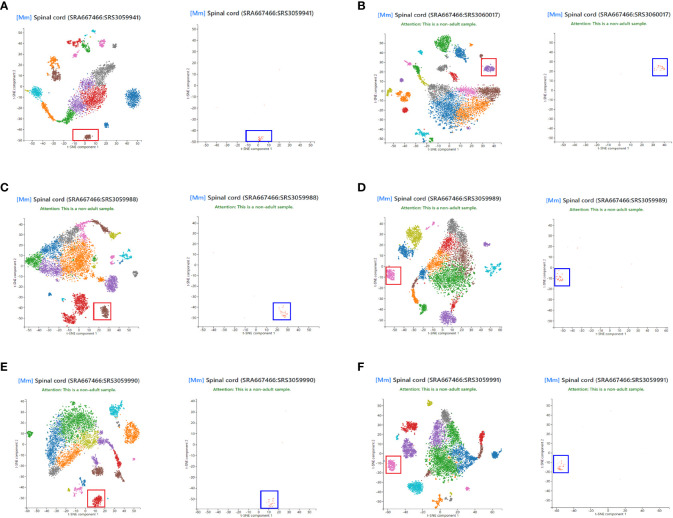
NLRP3 is exclusively expressed in disc microglia. Single cell expression profile for mouse spinal cord was obtained from Panglaodb. **(A–F)** In all analyzed 6 mouse spinal cord samples, NLRP3 (blue rectangle) was exclusively expressed in disc microglia clusters (red rectangle).

To assess the representativeness of NLRP3 in the total disc tissue compared to those in disc microglia, we obtained single cell expression profile for mouse spinal cord (Panglaodb), and examined the NLRP3 expression in different cell types in the disc. In all analyzed 6 samples, we found that NLRP3 was exclusively expressed in disc microglia clusters (**Figures 2A–F**). These data suggest that the patients’ data showing correlation between tissual NLRP3 and the pain or degeneration score was actually the correlation between disc microglia NLRP3 and the pain or degeneration score of the patients. Thus, NLRP3 levels in disc microglia correlate with the pain and disc degeneration level in LDD patients.

### Generation of microglia-specific NLRP3-KO or NLRP3-overexpressing mice

**Figure 3:**
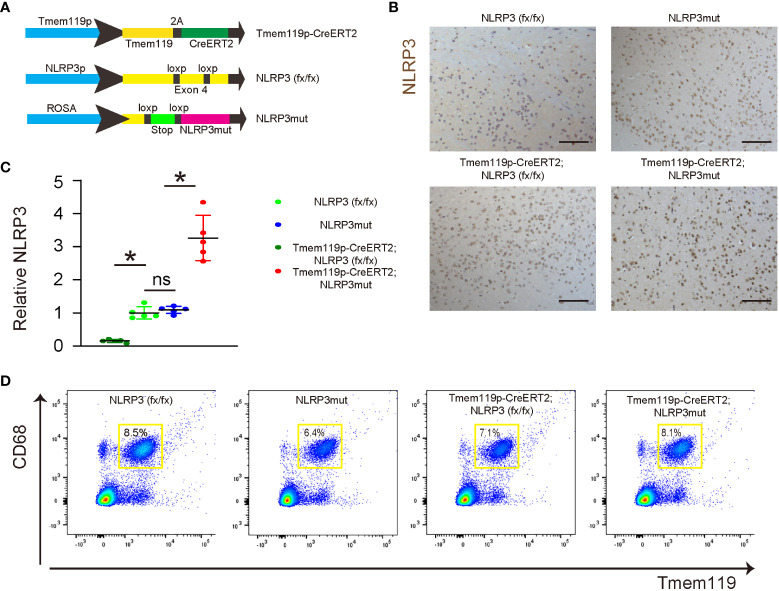
Generation of microglia-specific NLRP3-KO or NLRP3-overexpressing mice. **(A)** Illustration of mice with microglia-specific depletion of NLRP3 (Tmem119p-CreERT2; NLRP3 (fx/fx)) and their control NLRP3(fx/fx) mice, as well as mice with microglia-specific persistent expression of NLRP3 (Tmem119p-CreERT2; NLRP3mut) and their control NLRP3mut mice. **(B)** NLRP3 staining was done in spinal discs from tamoxifen-challenged mice. **(C, D)** Dissociated cells from spinal discs of the mice were FAC sorted for CD68+Tmem119+ microglia, the NLRP3 levels of which were checked by ELISA. **(C)** The relative levels to those from NLRP3(fx/fx) (=1) were shown. **(D)** The presentative flow charts of FACS sorting CD68+Tmem119+ microglia. *p<0.05. ns, non-significant. Scale bars are 100µm.

To assess the role of NLRP3 in microglia in LDD, we generated mice with microglia-specific depletion of NLRP3 (Tmem119p-CreERT2; NLRP3 (fx/fx)) and their control NLRP3(fx/fx) mice. We also generated mice with microglia-specific persistent expression of NLRP3 (Tmem119p-CreERT2; NLRP3mut) and their control NLRP3mut mice. At tamoxifen challenge, Tmem119p-CreERT2; NLRP3 (fx/fx) and Tmem119p-CreERT2; NLRP3mut mice developed microglia-specific NLRP3-KO or NLRP3-overexpressing. NLRP3 (fx/fx) and NLRP3mut mice at 15 weeks-old of age were used as wildtype controls (**Figure 3A**). NLRP3 staining was done in tamoxifen-challenged mice, showing loss of NLRP3 in discs from Tmem119p-CreERT2; NLRP3 (fx/fx) mice and increase in NLRP3 levels in discs from Tmem119p-CreERT2; NLRP3mut mice (**Figure 3B**). CD68+Tmem119+ cells (microglia) were sorted from spinal discs of the mice, confirming the expected alterations in NLRP3 levels in disc microglia (**Figures 3C, D**).

### NLRP3 depletion in microglia reduces phagocytosis potential and release of pro-inflammatory cytokines

**Figure 4:**
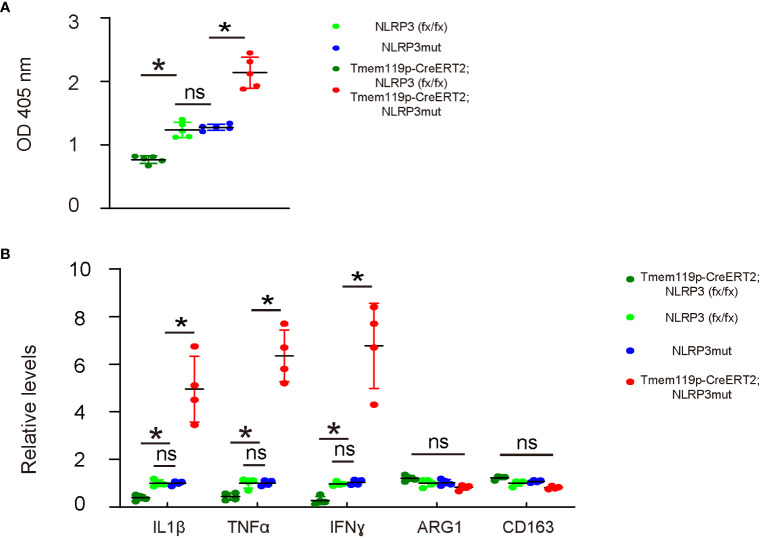
NLRP3 depletion in microglia reduces phagocytosis potential and release of pro-inflammatory cytokines. **(A)** Phagocytosis for zymosan was assessed in sorted disc microglia from mice with microglia-specific alteration in NLRP3 expression. **(B)** ELISA for IL-1β, TNFα, IFNɣ, ARG1 and CD163 in sorted disc microglia from mice with microglia-specific alteration in NLRP3 expression. The relative levels to those from NLRP3mut (=1) were shown. *p<0.05. ns, non-significant.

Phagocytosis is a major function of macrophages/microglia and is important for the neuroinflammation and subsequent disc degeneration. Thus, we examined phagocytosis in these mice with microglia-specific alteration in NLRP3 expression. A 0.5 hour’ zymosan intake showed significantly decreases in the phagocytosis of disc microglia from Tmem119p-CreERT2; NLRP3 (fx/fx) mice and significantly increases in the phagocytosis of disc microglia from Tmem119p-CreERT2; NLRP3mut mice (**Figure 4A**). Next, we analyzed some key factors related to microglia polarization and function. We detected significant reduction in IL-1β, TNFα and IFNɣ in disc microglia from Tmem119p-CreERT2; NLRP3 (fx/fx) mice and significantly increases in IL-1β, TNFα and IFNɣ in disc microglia from Tmem119p-CreERT2; NLRP3mut mice (**Figure 4B**). These factors are associated with pro-inflammatory functions. However, we did not detect any significant changes in two M2-microglia markers, ARG1 and CD163 (**Figure 4B**), suggesting that NLRP3 may regulate some functions of microglia but may not induce a complete polarization of microglia.

### NLRP3 depletion in microglia reduces disc degeneration and associated pain

**Figure 5:**
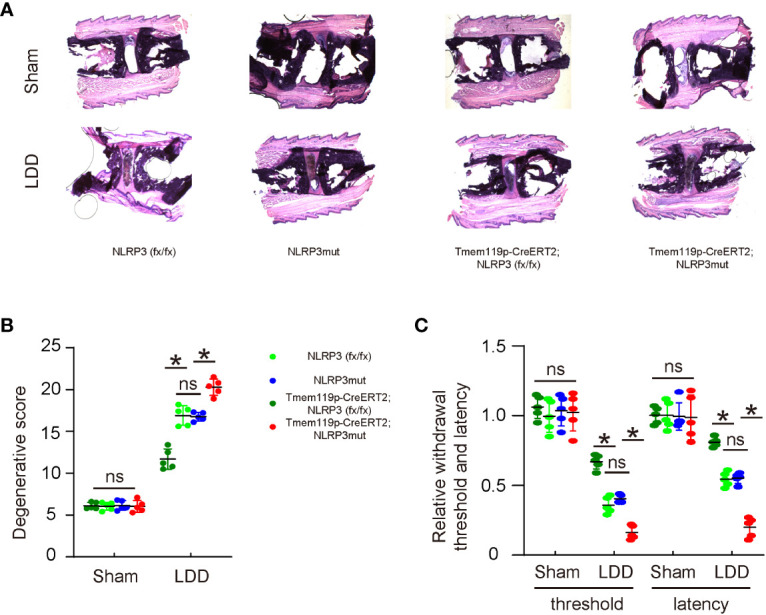
NLRP3 depletion in microglia reduces disc degeneration and associated pain. The effects of altering NLRP3 levels in microglia on disc degeneration and associated pain were examined in a mouse model for LDD. A total of 8 groups of mice were included in this experiment. Group 1, NLRP3 (fx/fx) mice received sham operation (Sham); Group 2: NLRP3 (fx/fx) mice received LDD induction (LDD); Group 3: NLRP3mut mice received sham operation; Group 4: NLRP3mut mice received LDD induction; Group 5, Tmem119p-CreERT2; NLRP3 (fx/fx) mice received sham operation; Group 6: Tmem119p-CreERT2; NLRP3 (fx/fx) mice received LDD induction; Group 7: Tmem119p-CreERT2; NLRP3mut mice received sham operation; Group 8: Tmem119p-CreERT2; NLRP3mut mice received LDD induction. Mice were analyzed 8 weeks after LDD or at age of 23-week-old. **(A, B)** Surgical induction of LDD and the quantification of disc degeneration were performed, shown by representative images **(A)** and by quantification for degenerative scores **(B)**. **(C)** A Von Frey filament test for pain evaluation, shown by the relative mechanically induced withdrawal threshold and by thermally induced withdrawal latency of the paw (normalized to those from NLRP3mut (=1)). *p<0.05. ns: no significance.

The effects of altering NLRP3 levels in microglia on disc degeneration and associated pain were examined in a mouse model for LDD. A total of 8 groups of mice were included in this experiment. Group 1, NLRP3 (fx/fx) mice received sham operation (Sham); Group 2: NLRP3 (fx/fx) mice received LDD induction (LDD); Group 3: NLRP3mut mice received sham operation; Group 4: NLRP3mut mice received LDD induction; Group 5, Tmem119p-CreERT2; NLRP3 (fx/fx) mice received sham operation; Group 6: Tmem119p-CreERT2; NLRP3 (fx/fx) mice received LDD induction; Group 7: Tmem119p-CreERT2; NLRP3mut mice received sham operation; Group 8: Tmem119p-CreERT2; NLRP3mut mice received LDD induction. Mice were analyzed 8 weeks after LDD or at age of 23-week-old. Surgical induction of LDD and the quantification of disc degeneration were performed, showing significant decreases in disc degeneration from Tmem119p-CreERT2; NLRP3 (fx/fx) mice and significant increases in disc degeneration from Tmem119p-CreERT2; NLRP3mut mice (**Figures 5A, B**). A Von Frey filament test was performed for pain assessment, showing significant improvement in mechanical induction in withdrawal threshold and significant improvement in thermal induction in withdrawal latency of the paw in Tmem119p-CreERT2; NLRP3 (fx/fx) mice as well as significant aggravation in mechanical induction in withdrawal threshold and significant aggravation in thermal induction in withdrawal latency of the paw in Tmem119p-CreERT2; NLRP3mut mice (**Figure 5C**). Together, these data suggest that NLRP3 depletion in microglia reduces disc degeneration and associated pain.

### Reduction in disc degeneration and associated pain by NLRP3 depletion in microglia may result from an alleviation of neuroinflammation

**Figure 6:**
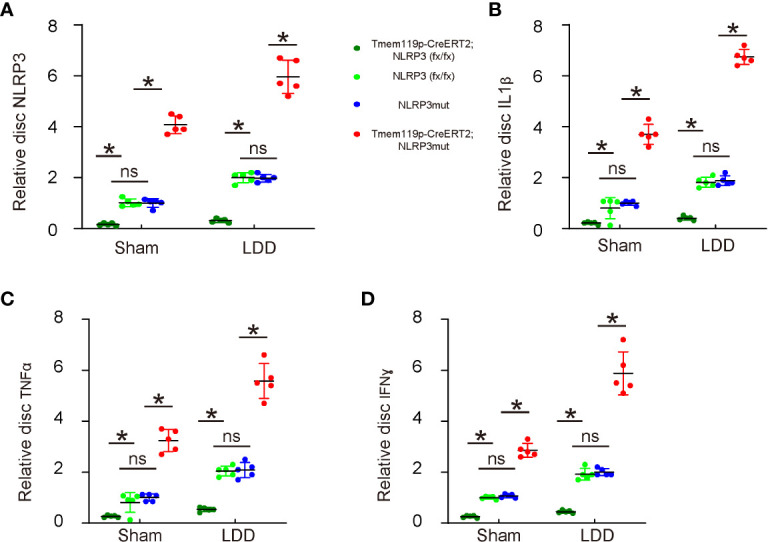
Reduction in disc degeneration and associated pain by NLRP3 depletion in microglia may result from an alleviation of neuroinflammation. **(A–D)** ELISA for NLRP3 **(A)**, IL-1β **(B)**, TNFα **(C)** and IFNɣ **(D)** levels in disc tissue sham/LDD-treated mice. The relative levels to those from NLRP3mut (=1) were shown. *p<0.05. ns, no significance.

Finally, the mechanism was explored. We analyzed levels of NLRP3 and several key pro-inflammatory factors in the mouse discs. First, we detected significant decreases in disc NLRP3 levels from Tmem119p-CreERT2; NLRP3 (fx/fx) mice and significant increases in disc NLRP3 levels from Tmem119p-CreERT2; NLRP3mut mice (**Figure 6A**), again validating the transgenic mice. Next, we detected significant decreases in disc IL-1β, TNFα and IFNɣ levels from Tmem119p-CreERT2; NLRP3 (fx/fx) mice and significant increases in disc IL-1β, TNFα and IFNɣ levels from Tmem119p-CreERT2; NLRP3mut mice (**Figure 6B–D**). These data suggest that reduction in disc degeneration and associated pain by NLRP3 depletion in microglia may result from an alleviation of neuroinflammation.

## Discussion

Activated microglia play a critical role in the development of LDD, which is a severe disease that causes neuropathic pain in affected people (2). IL-1β is a proinflammatory cytokine produced and secreted by activated microglia to induce the inflammation and the subsequent degradation of the disease discs (2). Recent findings suggest that cellular activation of IL-1β in cells typically needs the regulation by NLRP3-induced formation of inflammasome (14). However, the importance of NLRP3 in spinal microglia in LDD is not known and thus addressed in the current study.

It is known that the phenotypic adaptations of microglia during the initiation and progression of LDD are dynamic and involve not only changes in major functionality (M1 versus M2), but also some delicate changes in certain function such as phagocytotic, fibrotic, regenerative or else (18). Here in this study, we found that NLRP3 likely affects the polarization of microglia in such a delicate manner, since depletion of NLRP3 did not result in the upregulation of M2 marker ARG1 and CD163 (19), although it indeed alters the expression of some genes associated with the pro-inflammatory function of microglia. Importantly, the alteration in NLRP3 also resulted in altered phagocytosis of microglia. Since both exaggerated phagocytosis of microglia and augmentation of pro-inflammatory cytokines may induce severe inflammation and tissue damage (20), it is understandable that attenuation of these effects by NLRP3 KO could improve resolution of the inflammation and prevent LDD, while aggravation of these effects by permanent NLRP3 expression could increase the severity of the inflammation and promote LDD.

In the current study, we did not study the effects of altering NLRP3 in microglia on other immune cells, like macrophages or lymphocytes (21). Since a previous study has nicely shown that NLRP3 signaling in macrophages may regulate T cell differentiation and phenotypic changes from T helper type 1 cell to T helper type 2 cell (22). Future study may address the crosstalk between microglia and T cells through NLRP3 signaling in LDD.

To summarize, our data suggest that microglia-depletion of NLRP3 attenuates disc degeneration and reduces LDD-associated pain, while permanent activation of NLRP3 in microglia promotes disc degeneration and increases LDD-associated pain. Suppression of NLRP3 in microglia could be a promising strategy for LDD therapy.

## Data availability statement

The original contributions presented in the study are included in the article/Supplementary Material. Further inquiries can be directed to the corresponding author.

## Ethics statement

The studies involving human participants were reviewed and approved by Shanghai Jiao Tong University School of Medicine. The patients/participants provided their written informed consent to participate in this study. The animal study was reviewed and approved by Shanghai Jiao Tong University School of Medicine.

## Author contributions

PW and JZ are responsible for study conception and design, data acquisition and analysis. JZ wrote the manuscript and all authors have read the manuscript and agreed with the publication. JZ are responsible for funding and are the guarantee of the study. All authors contributed to the article and approved the submitted version.

## Funding

This work was supported by internal funding from XinHua Hospital.

## Conflict of interest

The authors declare that the research was conducted in the absence of any commercial or financial relationships that could be construed as a potential conflict of interest.

## Publisher’s note

All claims expressed in this article are solely those of the authors and do not necessarily represent those of their affiliated organizations, or those of the publisher, the editors and the reviewers. Any product that may be evaluated in this article, or claim that may be made by its manufacturer, is not guaranteed or endorsed by the publisher.
